# Relationships Between First-Year Student Resilience and Academic Stress

**DOI:** 10.3390/bs15060772

**Published:** 2025-06-03

**Authors:** David W. Nordstokke, Yvonne Hindes

**Affiliations:** School and Applied Child Psychology, Werklund School of Education, University of Calgary, Calgary, AB T2N 1N4, Canada

**Keywords:** academic stress, resilience, university students, transition to university

## Abstract

Academic stress is a prevalent issue among university students, with significant implications for mental health and academic performance. This exploratory study examined whether academic stress could be predicted from resilience sub-factors based on a three-factor model of resilience. An initial sample of 70 first-year university students completed self-report measures assessing mastery, relatedness, emotional reactivity, and academic stress. After accounting for missing data, 68 participants were female (65%; *n* = 44) and 35% (*n* = 24) were male. The mean age of the participants was 18.52 years, with a standard deviation of 1.26. Multiple regression analysis revealed that the sub-factors of mastery, relatedness, and reactivity were significant predictors of academic stress. Specifically, self-efficacy and perceived support were negatively associated with academic stress, and emotional sensitivity was positively associated with academic stress. The results have important implications for interventions aimed at reducing academic stress that focus on these resilience sub-factors could offer an effective approach for improving outcomes in transitioning students. Interventions such as cognitive training and mindfulness-based programs may strengthen students’ executive function difficulties, thereby improving their ability to cope with academic stress and foster resilience.

## 1. Introduction

Since the COVID-19 pandemic, enrolment in post-secondary education in Canada has continued to garner interest from both Canadian and international students, with Statistics Canada (2022) reporting a 2.6% increase in university enrolment for the 2019/2020 academic year as well as an increase in international (20.5%) and Canadian (7.5%) student graduates from university programs in 2021. Not only does post-secondary education offer both short- and long-term economic benefits, it has also been shown to be associated with other social and health benefits (Frenette, 2019; Oreopoulos & Petronijevic, 2013; Oreopoulos & Salvanes, 2011; Statistics Canada, 2022). Despite these advantages, attending post-secondary education also comes with heightened stress, the risk of mental health issues, and significant financial challenges (Auerbach et al., 2016; Duffy et al., 2020; Galarneau & Gibson, 2020; Linden et al., 2023), which can further impact academic performance and overall well-being (Auerbach et al., 2016; Pozos-Radillo et al., 2014; C. Zhang et al., 2022). The ongoing formation of personal, social, and occupational identities during this period amplifies the stress and vulnerability experienced by students (Arnett, 2015). The pervasive impact of academic stress highlights the importance of students developing effective coping strategies (Zarei et al., 2016).

Studying students who manage to function well despite these challenges provides important insight into the factors that contribute to resilience during post-secondary education. Resilience, defined as the ability to thrive in the face of adversity, challenge, or risk, has been extensively studied in contexts of severe adversity (Masten, 2014). However, resilience is also relevant to more ordinary life experiences, such as post-secondary education, which demands adaptations to significant changes, increased independence, and goal-directed behavior. Investigating resilience factors and academic stress may provide valuable insight, providing a better understanding of these relationships among university students.

### 1.1. The Literature

#### 1.1.1. Academic Stress

Although post-secondary education has been associated with positive growth across multiple domains of a student’s life (Frenette, 2019; Oreopoulos & Petronijevic, 2013; Oreopoulos & Salvanes, 2011), students in post-secondary education also face a wide variety of stressors, especially first-year students transitioning to university, which can play a vital role in their learning and academic achievements, interpersonal relationships, and well-being (Auerbach et al., 2016; Duffy et al., 2020; Linden et al., 2023; Pozos-Radillo et al., 2014; Robotham, 2008; Smathers et al., 2024; C. Zhang et al., 2022). According to the Spring 2022 Canadian Reference Group data from the American College Health Association’s National College Health Assessment (ACHA-NCHA III), 72.5% of students reported experiencing moderate or high levels of stress within the last 30 days, and 34.2% indicated that stress had negatively impacted their academic performance (American College Health Association, 2022). Recent research showed that Canadian post-secondary students reported rates of 48.69% for stress in 2020 and rates of 56.88% for stress in 2021 (Dubale et al., 2024), suggesting that stress levels are increasing among university students. A scoping review by Linden and Stuart (2020) also found student stress and distress to be a common theme amongst post-secondary students.

Academic stress in university students leads to several negative outcomes, significantly impacting their mental health and academic performance. High levels of perceived stress are strongly associated with lowered mental well-being, with academic pressure being a primary contributor (Slimmen et al., 2022). This stress can manifest as emotional exhaustion and depression, which are exacerbated by factors such as workload and work complexity (Reichel et al., 2024). Similarly, academic and familial stress have been shown to directly contribute to depression, negatively affecting students’ learning outcomes (Deng et al., 2022). Moreover, stress related to adapting to university life is linked to psychiatric morbidity, including depression and somatic disorders, with these effects being more pronounced than those from childhood trauma or current family issues (Y. Zhang et al., 2022). This growing concern highlights the need for effective support systems and interventions that can help students manage their stress and improve their overall mental health during their academic journey. As universities continue to grapple with this issue, implementing comprehensive mental health resources and promoting a culture of openness around stress management will be crucial in fostering a supportive environment for students.

#### 1.1.2. Resilience

A common definition of resilience is that of “ordinary magic” (Aburn et al., 2016; Masten, 2015), meaning that resilience arises from ordinary adaptational processes and includes systems such as cognitive functioning, family, and community. Within this framework, resilience is described as a common construct involving human adaptational systems and responses to adversity (Janssen et al., 2011; Masten, 2001). Masten’s (2001) definition emphasizes resilience as both a process and an outcome, reflecting the ability of individuals or systems to maintain or regain functionality in the face of adversity. It also describes the important and crucial role of adaptations for resilience.

In relation to students, resilience is demonstrated and observed when students can use their adaptational systems (i.e., support systems, drive/motivation, intellectual and cognitive skills) to overcome the adversities and challenges frequently associated with and experienced during their post-secondary education. For example, a study by Versteeg and Kappe (2021) on a large sample of higher education students during the COVID-19 pandemic revealed that resilience served as a protective factor that mediated the positive direct effect of perceived academic stress on depression, demonstrating the importance of understanding resilience factors. Further, Chen et al. (2022) found that resilience was a significant mediator in the relationship between psychological distress and academic burnout, illustrating the importance of resilience for academic outcomes. While these studies included resilience as a unitary construct or trait, which has provided valuable insight into the topic, it is important to consider resilience as a multidimensional construct. Considering this complexity may offer a more nuanced understanding of the role of resilience in managing academic stress.

Prince-Embury built on Masten’s conceptualization of resilience by developing a three-factor model of personal resilience (Prince-Embury, 2006, 2007, 2014): (a) sense of mastery, which consists of optimism, self-efficacy, and adaptability (Prince-Embury, 2014; Prince-Embury et al., 2017); (b) sense of relatedness, which consists of trust of others, tolerance of others, perceived support, and comfort with others (Prince-Embury, 2014; Prince-Embury et al., 2017); and (c) emotional reactivity, which consists of sensitivity to stressors, level of impairment, and emotional recovery (Prince-Embury, 2014; Prince-Embury et al., 2017). What makes this model particularly compelling is its ability to distill the complex construct of resilience into a concise and coherent framework while maintaining comprehensive coverage.

The three-factor model simplifies resilience by breaking it down into fundamental components, facilitating easier operationalization and interpretation. Although it simplifies resilience, the model remains comprehensive by incorporating the numerous adaptational systems identified in resilience research. Additionally, the model is linked to well-validated and psychometrically sound measures of resilience, such as the Resilience Scale for Children and Adolescents (RSCA; Prince-Embury, 2006, 2007) and the Resiliency Scale for Young Adults (RSYA; Prince-Embury et al., 2017). Unlike other resilience measures that often focus narrowly on specific components, the RSCA and RSYA offer a broad assessment of resilience-related constructs and exhibit reliable factor structures and validity. For these reasons, this model was employed as the theoretical framework in the current study.

#### 1.1.3. Current Study

While resilience is often characterized as the capacity to adapt successfully in the face of stressors or adversity, academic stress remains a prevalent challenge, particularly among post-secondary students (Linden et al., 2023; Pozos-Radillo et al., 2014). Research has consistently shown that academic stress negatively impacts mental health, academic performance, and overall well-being (Auerbach et al., 2016; Pascoe et al., 2020; Pozos-Radillo et al., 2014; C. Zhang et al., 2022).

The purpose of the current study is to explore the nature of the relationship between resilience factors (mastery, relatedness, and reactivity) and academic stress through four research questions:

1. To what extent do resilience factors (i.e., mastery, relatedness, and reactivity) correlate with each other?
2. To what extent do mastery sub-factors (i.e., optimism, adaptability, and self-efficacy) predict academic stress?
3. To what extent do relatedness sub-factors (i.e., trust, tolerance, comfort, and support) predict academic stress?
4. To what extent do reactivity sub-factors (i.e., sensitivity, impairment, and recovery) predict academic stress?

## 2. Methods

### 2.1. Participants and Recruitment

Upon attaining institutional ethics approval, first-year undergraduate students were recruited at a research-intensive Western Canadian university utilizing a convenience sampling approach. While the specific ethnicities of the participants were not collected in the current study, the University of Calgary (2020) reported that 2.8% of students identified as Indigenous, 29.1% were racialized minorities, and 68.1% identified as white. Participants were eligible to participate in the study if they were in their first year of post-secondary studies and had never been previously enrolled in post-secondary studies. Participants were recruited via a presentation in several first-year classes, which included a brief rationale and description of the study and participant eligibility criteria. Interested students were then invited to participate in the study via a link to a secure online survey platform where they provided informed consent and completed the survey. Each participant was randomly assigned a participant number to ensure anonymity and their data were stored on a password-protected drive locked in a filing cabinet.

Seventy participants completed the study; however, two participants were removed from the dataset and were not included in the analyses because they were missing a significant amount of data (>50% of their data). Of the remaining participants (*n* = 68), the majority identified as female (65%; *n* = 44) and the remaining 35% (*n* = 24) identified as male. The mean age of the participants was 18.52 years, with a standard deviation of 1.26. Students were sampled from a variety of different university programs. The most reported programs of study were Biological Sciences (25%), Engineering (19%), Psychology (15%), Computer Science (9%), and Astrophysics/Physics (7%). Other programs were represented by fewer than 5% of participants in the study. This variable was not included in the primary analyses.

### 2.2. Measures

#### 2.2.1. College Student Stress Scale (CSSS)

The CSSS (Feldt, 2008) is a short, 11-item scale used to measure student stress; however, based on the results of a confirmatory factor analysis (Feldt & Koch, 2011), which showed that only 7 of the 11 items had an adequate fit, this study used the recommended 7-item scale instead of the original 11-item scale. Each of the questions on the CSSS is rated using a 5-point Likert scale and the total score is derived by summing together the ratings on the seven questions. Research (Feldt, 2008; Feldt & Koch, 2011) has supported the single-factor model, the internal consistency of the CSSS (*α* = 0.82), the test–retest reliability over a 5-week period (*r* = 0.62 to *r* = 0.86), and the convergent validity with the perceived stress scale (Cohen et al., 1983). For the current study sample, the coefficient alpha was 0.88.

#### 2.2.2. Resiliency Scale for Young Adults (RSYA)

The RSYA (Prince-Embury et al., 2017), which is a modification of the Resiliency Scales for Children and Adolescents (Prince-Embury, 2006, 2007), consists of 50 items, rated on a 5-point Likert scale, and is used to measure different factors of trait resilience in young adults. The RSYA scores are calculated by summing the items in each of the subscales and provides an evaluation of an adult’s sense of mastery (comprising optimism, adaptability, and self-efficacy), sense of relatedness (comprising trust of others, tolerance of others, perceived support, and comfort with others), and emotional reactivity (comprising sensitivity to stressors, level of impairment, and emotional recovery); the scores for each of these factors is derived by summing together the scores for the items connected to each domain. A confirmatory factor analysis by Prince-Embury et al. (2017) using a university sample supported the three-factor model over a one- or two-factor model. Their research has also shown that the RSYA has acceptable internal consistency for the three factors (ranging from *α* = 0.89 to *α* = 0.92) as well as demonstrating convergent and divergent validity with respect to satisfaction with life, psychological flourishing, emotional intelligence, anxiety, stress, and depression. For the current study sample, the coefficient alphas were as follows: mastery subscale = 0.82, relatedness subscale = 0.90, and reactivity subscale = 0.93.

#### 2.2.3. Procedure

Once the research ethics board approval for this study was obtained, the participants were recruited. The research team contacted professors of undergraduate courses to receive permission to visit their lectures to invite students to participate in the research. With consent from the professors, the researcher invited undergraduate students to participate during lectures. After participants indicated their interest in participating in the study, they were provided with a link to the online survey platform, where they provided their informed consent and completed the demographics questions and the questionnaires.

### 2.3. Data Preparation

Missing data were addressed via listwise deletion, as the two cases that were identified as having missing values were missing more than 50% of their data, leaving (*n* = 68) participants for the analysis. There were no outliers present in the data, as verified via the use of boxplots and the Mahalanobis distance. Skew values in conjunction with the Shapiro–Wilks test were used to investigate whether the distributions followed a normal form. Skew values ranged between −0.66 to 0.45 and the results of the Shapiro–Wilks test across academic stress, mastery, relatedness, and reactivity were non-significant, indicating that they were distributed approximately normally.

### 2.4. Correlational Analysis

Bivariate Pearson correlations were computed for the resilience factor scores.

### 2.5. Regression Analysis

To explore whether any of the sub-factors for each of the three resilience factors (mastery, relatedness, and reactivity) predicted academic stress, three multiple regression analyses were employed. In each of the three models, variables were included using the enter method. Power analysis using G*Power 3.1.9.7 indicated that, using the values (*f*^2^ = 0.19; α = 0.05; power = 0.80), a sample size of 68 was adequate for conducting the analysis. The regression analyses were performed using R 4.4.1 (R Core Team, 2024).

## 3. Results

### 3.1. Descriptive and Preliminary Analyses

The following section summarizes the descriptive statistics for academic stress, mastery, reactivity, and relatedness, stratified by gender. Table 1 presents the sample sizes, means, standard deviations (SDs), minimum and maximum scores, and skewness values for females and males. Preliminary analyses include independent sample *t*-tests to analyze gender differences and Pearson correlations to investigate the associations among the variables in the study.

Female participants’ (*n* = 44) reported levels of academic stress were (M = 24.23, SD = 5.19) and for male participants, they were (*n* = 24; M = 21.92, SD = 6.47). Scores ranged from 12 to 33 for females and from 12 to 34 for males. The skewness values for both genders indicated a near-normal distribution (−0.22 for females, 0.30 for males). Mastery scores for females were (M = 55.84, SD = 6.81) and for males were (M = 55.65, SD = 8.70). For females, scores ranged from 41 to 69, while for males, scores ranged from 32 to 70. Both distributions were slightly negatively skewed (−0.52 for females, −0.79 for males). Females (M = 72.50, SD = 10.79) and males (M = 71.92, SD = 10.82) reported comparable levels of relatedness. Scores ranged from 46 to 97 for females and from 52 to 97 for males. The skewness values (−0.16 for females, 0.29 for males) indicated a relatively symmetric distribution. Female participants’ reactivity scores were (M = 38.45, SD = 9.23) and males’ scores were (M = 33.55, SD = 10.15). Females’ scores ranged from 20 to 57, while males’ scores ranged from 18 to 55. The skewness values (0.22 for females, 0.45 for males) suggest a slight positive skew for both genders. Independent sample *t*-tests using a Bonferroni-corrected alpha (*α* = 0.05/4 = 0.0125) revealed that there were no significant differences in gender across academic stress, mastery, relatedness, and reactivity; thus, gender was not included in the regression analyses. In addition, there were no significant (*p* < 0.05) correlations between age, academic stress, and the resilience variables; therefore, age was not considered in the regression analysis.

### 3.2. Correlational Analysis

Correlations among mastery, relatedness, and reactivity are presented in Table 2.

Mastery was positively associated with relatedness (*r* = 0.66, *p* < 0.001) but negatively correlated with reactivity (*r* = −0.41, *p* < 0.001). Relatedness demonstrated a strong positive correlation with mastery (*r* = 0.66, *p* < 0.001) and a negative correlation with reactivity (*r* = −0.33, *p* < 0.01). Reactivity was negatively associated with mastery (*r* = −0.41, *p* < 0.001) and relatedness (*r* = −0.33, *p* < 0.01).

### 3.3. Mastery Sub-Factor Regression Analysis

In assessing the assumptions for the regression, the Durbin–Watson value was 2.035, supporting the independence of the data. The variance inflation factors for each predictor were within acceptable values (optimism = 1.71; self-efficacy = 1.55; adaptability = 1.82), demonstrating that there was no evidence of multicollinearity. Visual inspection of residual plots revealed a linear relationship and homoscedasticity. Mahalanobis distances were used (df = 3) to check for multivariate outliers; none were present. The overall regression was significant: *F* (3, 64) = 15.09, *p* < 0.001, with an adjusted R^2^ = 0.39; *f*^2^ = 0.69. See Table 3 for parameter estimates.

### 3.4. Relatedness Sub-Factor Regression Analysis

In assessing assumptions for the regression, the Durbin–Watson value was 2.15, supporting the independence of the data. The variance inflation factors for each predictor were within acceptable values (comfort = 1.69; trust = 1.83; tolerance = 1.65; support = 1.72), demonstrating that there was no evidence of multicollinearity. Visual inspection of residual plots revealed a linear relationship and homoscedasticity. Mahalanobis distances were used (df = 4) to check for multivariate outliers; none were present. The overall regression was significant: *F* (4, 63) = 3.98, *p* = 0.006, with an adjusted R^2^ = 0.15; *f*^2^ = 22. Parameter estimates are listed in Table 4.

### 3.5. Reactivity Sub-Factor Regression Analysis

In assessing the assumptions for the regression, the Durbin–Watson value was 2.21, supporting the independence of the data. The variance inflation factors for each predictor were within acceptable values (sensitivity = 2.49; impairment = 1.90; recovery = 2.17), demonstrating that there was no evidence of multicollinearity. Visual inspection of residual plots revealed a linear relationship and homoscedasticity. Mahalanobis distances were used (df = 3) to check for multivariate outliers; none were present. The overall regression was significant: *F* (3, 64) = 11.06, *p* < 0.001, with an adjusted R^2^ = 0.31; *f*^2^ = 0.46. See Table 5 for parameter estimates.

## 4. Discussion

The present study aimed to investigate the predictive relationship between the resilience sub-factors of mastery, relatedness, and reactivity and academic stress.

### 4.1. Resilience and Academic Stress

Resilience has long been conceptualized as the capacity to adapt successfully in the face of stress or adversity, grounded in the presence of individual and contextual protective factors that buffer against negative outcomes (Rutter, 1987; Masten, 2001). Classic resilience frameworks emphasize that resilience is not a fixed trait but rather the product of dynamic interactions between risk exposure and protective processes. Protective factors are understood as characteristics or conditions that moderate the effects of stressors and promote positive adaptation, particularly under high-risk conditions (Rutter, 1987). In the context of academic stress among university students, this study identified three resilience-related constructs, self-efficacy, perceived support, and emotional sensitivity, that align with this framework in theoretically meaningful ways. Self-efficacy and perceived support represent prototypical protective factors, with the former reflecting internal beliefs in one’s competence to manage academic demands (Bandura, 1997), and the latter reflecting external relational resources that can buffer the impact of academic challenges (Cohen & Wills, 1985). In contrast, emotional sensitivity is better conceptualized as a vulnerability factor; a dispositional tendency toward heightened emotional reactivity that may amplify stress responses when not moderated by adequate protective mechanisms (Masten & Garmezy, 1985). Taken together, these factors illustrate the classic resilience framework’s core proposition: that adaptive outcomes in the face of stress depend not only on the presence of risk, but on the availability and efficacy of specific protective processes that modulate the individual’s response.

### 4.2. Mastery Sub-Factors and Academic Stress

The analysis revealed that of the mastery sub-factors, self-efficacy was significantly and negatively associated with academic stress, accounting for about 39% of the variance in academic stress and indicating a strong relationship. Students with higher levels of self-efficacy reported lower levels of stress, which is consistent with the notion that individuals who feel more in control and confident in their academic environment are better able to manage stressors (Kennett et al., 2021). Neither optimism nor adaptability were significant in the model. This lack of significance may be attributed to several factors. It is possible that these variables do not have a direct effect on academic stress, or that their effects may be mediated through other variables not included in our model. Understanding the role of self-efficacy in the context of academic stress may be interpreted through Lazarus and Folkman’s (1984) Transactional Model of Stress and Coping which posits that stress is impacted by how individuals appraise situations and their perceived coping resources. Self-efficacy influences secondary appraisals, as students who believe they can manage academic tasks perceive stressors as less impactful, thereby reducing the impact of the stressors. Bandura’s (1997) self-efficacy theory further clarifies how beliefs in one’s personal competence reduce stress responses, as students with high self-efficacy engage in more adaptive coping strategies and interpret academic setbacks less catastrophically. This protective effect is supported by findings that self-efficacy buffers stress responses and enhances resilience (Schueler et al., 2021). Individuals with high self-efficacy are more likely to set challenging goals and remain committed to achieving them, thereby reducing stress linked to self-doubt (Artino, 2012). Moreover, self-efficacious students are more likely to engage in proactive behaviors, such as seeking help or managing their time effectively, which further alleviates stress. For instance, a digital self-efficacy training program was shown to reduce hopelessness and anxiety while increasing self-efficacy among stressed university students, suggesting that self-efficacy can help mitigate stress-related outcomes (Rohde et al., 2024). This highlights the importance of fostering self-efficacy in educational settings, as it not only enhances academic performance but also promotes overall mental well-being among students.

### 4.3. Relatedness Sub-Factors and Academic Stress

In this study, we also examined the predictive relationships between the four relatedness sub-factors (i.e., comfort, trust, tolerance, and support) and academic stress. Our regression model accounted for 15% of the variance in academic stress, indicating that these factors collectively have a modest impact on students’ stress levels. Among the predictors, only support emerged as a significant factor, inversely associated with academic stress. This suggests that higher levels of perceived support are linked to lower levels of academic stress. This finding aligns with the existing literature emphasizing the critical role of support in influencing such outcomes (Clark et al., 2024; McLean et al., 2023). Cohen and Wills’ (1985) Stress-Buffering Hypothesis posits that the presence of supportive relationships does not merely enhance general well-being but becomes especially critical during periods of high stress. When academic pressures intensify, students who perceive strong support from peers, family, or faculty may reappraise stressors as more manageable, thus experiencing lower physiological and emotional stress responses. Pinto et al. (2024) emphasize the importance of promoting inclusive student communities and enhancing student well-being through interactions that foster a sense of belonging. Implementing social support systems like peer mentoring, faculty engagement, and community-building initiatives can significantly contribute to this goal. By facilitating meaningful connections among and between students and faculty, universities can create a supportive academic environment that sustains feelings of social inclusion and belonging beyond the initial weeks of university life.

### 4.4. Reactivity and Academic Stress

Reactivity accounted for 31% of the variance in academic stress, suggesting a strong relationship. Of the reactivity sub-factors included in the regression analysis, sensitivity was significantly and positively associated with academic stress, indicating that students who were more emotionally sensitive experienced higher stress levels. Sensitivity, defined as the intensity of one’s response to stressors, was positively related to academic stress, suggesting that highly sensitive students may perceive academic challenges as being more negative or overwhelming. Emotional sensitivity represents a distinct vulnerability factor within models of stress and resilience. In the Transactional Model of Stress and Coping (Lazarus & Folkman, 1984), sensitivity influences the primary appraisal process, whereby individuals assess whether a situation is threatening. Students with heightened emotional sensitivity may appraise routine academic tasks as more threatening than they objectively are, resulting in elevated stress levels. This pattern aligns with research linking high emotional reactivity to increased threat appraisals and decreased emotional regulation (Spătaru & Maricuțoiu, 2024). This sensitivity can lead to feelings of being overwhelmed by sensory and emotional stimuli, as highlighted in interviews with individuals who identify as highly sensitive, where they reported relief upon the self-attribution of this trait, yet also noted the negative impact of being easily overwhelmed (Roth et al., 2023). This heightened stress perception can lead to the overactivation of emotional and physiological stress responses, even in relatively low-stakes situations. For instance, a sensitive student may catastrophize routine academic setbacks, such as receiving critical feedback, which can increase anxiety and reduce problem-solving capacity.

### 4.5. Implications and Future Research

The findings of this study suggest that specific dimensions of resilience are differentially associated with academic stress among university students. Self-efficacy demonstrated a large and negative association with academic stress, consistent with the idea that strong beliefs in one’s academic competence are central to adaptive stress management. Perceived support also showed a moderate negative association with academic stress, reinforcing the view that social resources are important, though perhaps secondary to internal competence beliefs. In contrast, emotional sensitivity was moderately and positively associated with academic stress, indicating that heightened emotional reactivity may increase vulnerability rather than promote resilience in demanding academic environments. Together, these results support the view that resilience is not a singular protective factor but a constellation of characteristics with context-dependent effects.

These findings should be interpreted cautiously given the modest sample size and cross-sectional design, but they suggest important directions for future research. The results highlight the need for theoretical models of academic resilience to distinguish between dimensions that reliably buffer stress and those that may, under certain conditions, exacerbate it. Future studies should examine how competence-based factors, relational support, and emotional reactivity interact over time to shape students’ experiences of academic stress. Longitudinal and multisite designs would be particularly valuable in testing whether the observed patterns are stable across different academic settings and populations. More broadly, this study underscores the value of adopting a differentiated view of resilience that accounts for both protective and vulnerability processes in understanding students’ adaptation to academic demands. The exploratory nature of these findings underscores the importance of cautious interpretation in resilience research. Given the sample size and the analytical strategy employed, these results are best viewed as preliminary evidence of differential patterns rather than definitive statements about causal relationships. Future research employing larger samples, longitudinal designs, and replication across diverse educational settings is necessary to establish the robustness and generalizability of the observed associations.

### 4.6. Study Limitations

There are several limitations that should be noted. First, the relatively small sample size (*n* = 68) limits the generalizability of the findings of the regression analyses. While the sample size was minimally sufficient for the statistical analyses, larger samples would provide a more stable estimation of the parameters and increase the power to detect smaller effects to avoid possibly overfitting the models. Separate regression models were conducted for each set of sub-factors to examine their individual contributions to academic stress. While this approach allowed for a more focused investigation of each resilience dimension, it also increased the risk of Type I errors. These analytic decisions should be considered when interpreting the findings. Also, this study did not account for possible confounders (e.g., prior mental health conditions). The use of a single-site convenience sample further restricted the external validity and limited the applicability of the findings beyond this institutional context. The ability to make causal inferences or broad generalizations about the associations between the variables was absent due to the cross-sectional design of the study. In addition, the study relied on self-reported measures of resilience factors and academic stress, which may be subject to biases such as social desirability and recall bias. These limitations are particularly salient in research on stress and psychological traits, where self-perceptions may not fully capture behavioral or physiological indicators of resilience. Future research would benefit from incorporating objective measures such as behavioral tasks, peer ratings, or biological indicators to provide a more comprehensive assessment of student stress responses and resilience processes over time.

## 5. Conclusions

This study provides preliminary evidence that specific dimensions of resilience, particularly self-efficacy, perceived support, and emotional sensitivity, are differentially associated with academic stress among university students. The findings highlight the importance of viewing resilience as a multidimensional construct rather than as a unitary construct. Although the results are exploratory and based on a modest sample size, they suggest that competence beliefs and relational resources may serve as important buffers against stress, while heightened emotional sensitivity may function as a vulnerability. Future research using larger samples and longitudinal designs is needed to confirm these patterns and further clarify the dynamic roles of resilience-related factors in academic adaptation. These findings offer an initial step toward a more differentiated understanding of resilience processes in higher education contexts.

## Figures and Tables

**Table 1:** Descriptive statistics.

		Gender	N	Mean	SD	Minimum	Maximum	Skewness
Academic Stress		Female	44	24.23	5.19	12	33	−0.22
		Male	24	21.92	6.47	12	34	0.3
Mastery		Female	44	55.84	6.81	41	69	−0.52
		Male	24	55.65	8.7	32	70	−0.79
Reactivity		Female	44	38.45	9.23	20	57	0.22
		Male	24	33.55	10.15	18	55	0.45
Relatedness		Female	44	72.5	10.79	46	97	−0.16
		Male	24	71.92	10.82	52	97	0.29

**Table 2:** Correlation table.

	Mastery	Reactivity	Relatedness
Mastery	—		
Reactivity	−0.41 ***	—	
Relatedness	0.66 ***	−0.33 **	—

Note. ** *p* < 0.01, *** *p* < 0.001.

**Table 3:** Model parameter estimates for regression analysis of mastery subscales.

Predictor	Estimate	SE	95% CI Lower	95% CI Upper	*β*	t	*p*	*η* ^2^ *p*
Intercept	23.41	0.54	22.32	24.5	0	43.01	<0.001	
OPT	−0.14	0.23	−0.6	0.33	−0.07	−0.6	0.554	0.006
EFF	−1.28	0.23	−1.74	−0.82	−0.66	−5.55	<0.001	0.33
ADA	0.23	0.26	−0.29	0.75	0.11	0.87	0.387	0.01

For each of the predictors, the coefficients were as follows: *β* = −0.07, *p* > 0.05 for optimism; *β* = −0.66, *p* < 0.001 for self-efficacy; and *β* = 0.11, *p* > 0.05 for adaptability.

**Table 4:** Model parameter estimates for regression analysis of relatedness subscales.

Predictor	Estimate	SE	95% CI Lower	95% CI Upper	*β*	t	*p*	*η* ^2^_p_
Intercept	23.41	0.64	22.13	24.69	0	36.54	<0.001	
Comfort	−0.12	0.24	−0.59	0.36	−0.07	−0.49	0.625	0.004
Trust	−0.18	0.23	−0.65	0.29	−0.12	−0.77	0.444	0.009
Tolerance	0.25	0.34	−0.43	0.92	0.11	0.73	0.47	0.008
Support	−0.61	0.23	−1.07	−0.15	−0.39	−2.63	0.011	0.1

For each of the predictors, the coefficients were as follows: *β* = −0.07, *p* > 0.05 for comfort; *β* = −0.11, *p* > 0.05 for trust; *β* = 0.11, *p* > 0.05 for tolerance; and *β* = −0.39, *p* < 0.05 for support.

**Table 5:** Regression model parameter estimates for reactivity subscales.

Predictor	Estimate	SE	95% CI Lower	95% CI Upper	*β*	t	*p*	*η* ^2^_p_
Intercept	23.41	0.58	22.26	24.57	0	40.55	<0.001	
Sensitivity	0.56	0.24	0.09	1.04	0.38	2.36	0.021	0.08
Impairment	0.34	0.24	−0.14	0.82	0.2	1.42	0.161	0.03
Recovery	0.1	0.22	−0.33	0.54	0.07	0.48	0.633	0.01

For each of the predictors, the coefficients were as follows: *β* = 0.38, *p* = 0.02 for sensitivity; *β* = 0.20 *p* > 0.05 for impairment; and *β* = 0.07, *p* > 0.05 for recovery.

## Data Availability

The data supporting the findings of this study are not publicly available due to ethical restrictions imposed by the Conjoint Faculties Research Ethics Board (CFREB) at the University of Calgary. De-identified data may be made available from the corresponding author upon reasonable request and with appropriate institutional approval.
